# IS*1*-related large-scale deletion of chromosomal regions harbouring the oxygen-insensitive nitroreductase gene *nfsB* causes nitrofurantoin heteroresistance in *Escherichia coli*

**DOI:** 10.1099/mgen.0.001102

**Published:** 2023-09-06

**Authors:** Yu Wan, Akshay Sabnis, Zaynab Mumin, Isabelle Potterill, Elita Jauneikaite, Colin S. Brown, Matthew J. Ellington, Andrew Edwards, Shiranee Sriskandan

**Affiliations:** ^1^​ NIHR Health Protection Research Unit in Healthcare Associated Infections and Antimicrobial Resistance, Department of Infectious Disease, Imperial College London, London, UK; ^2^​ HCAI, Fungal, AMR, AMU and Sepsis Division, UK Health Security Agency, London, UK; ^3^​ Centre for Bacterial Resistance Biology, Imperial College London, London, UK; ^4^​ Reference Services Division, National Infection Service, UK Health Security Agency, London, UK; ^5^​ Department of Infectious Disease Epidemiology, School of Public Health, Imperial College London, London, UK; ^6^​ MRC Centre for Global Infectious Disease Analysis, School of Public Health, Imperial College London, London, UK

**Keywords:** comparative genomics, *Escherichia coli*, insertion sequences, nitrofurantoin heteroresistance, population analysis profiling, whole-genome sequencing, structural variation, IS1 elements, resistance mechanism, hybrid genome assembly

## Abstract

Nitrofurantoin is a broad-spectrum first-line antimicrobial used for managing uncomplicated urinary tract infection (UTI). Loss-of-function mutations in chromosomal genes *nfsA, nfsB* and *ribE* of *Escherichia coli* are known to reduce nitrofurantoin susceptibility. Here, we report the discovery of nitrofurantoin heteroresistance in *E. coli* clinical isolates and a novel genetic mechanism associated with this phenomenon. Subpopulations with lower nitrofurantoin susceptibility than major populations (hereafter, nitrofurantoin-resistant subpopulations) in two *E. coli* blood isolates (previously whole-genome sequenced) were identified using population analysis profiling. Each isolate was known to have a loss-of-function mutation in *nfsA*. From each isolate, four nitrofurantoin-resistant isolates were derived at a nitrofurantoin concentration of 32 mg l^−1^, and a comparator isolate was obtained without any nitrofurantoin exposure. Genomes of derived isolates were sequenced on Illumina and Nanopore MinION systems. Genetic variation between isolates was determined based on genome assemblies and read mapping. Nitrofurantoin minimum inhibitory concentrations (MICs) of both blood isolates were 64 mg l^−1^, with MICs of major nitrofurantoin-susceptible populations varying from 4 to 8 mg l^−1^. Two to 99 c.f.u. per million demonstrated growth at the nitrofurantoin concentration of 32 mg l^−1^, which is distinct from that of a homogeneously susceptible or resistant isolate. Derived nitrofurantoin-resistant isolates had 11–66 kb deletions in chromosomal regions harbouring *nfsB*, and all deletions were immediately adjacent to IS*1*-family insertion sequences. Our findings demonstrate that the IS*1*-associated large-scale genetic deletion is a hitherto unrecognized mechanism of nitrofurantoin heteroresistance and could compromise UTI management. Further, frequencies of resistant subpopulations from nitrofurantoin-heteroresistant isolates may challenge conventional nitrofurantoin susceptibility testing in clinical settings.

## Data Summary

Whole-genome sequencing reads and genome assemblies generated in this study have been deposited under BioProject PRJEB58678 in the European Nucleotide Archive (ENA). Accession numbers are listed in Table S1, available in the online version of this article. Previously generated Illumina whole-genome sequencing reads of progenitor isolates EC0026B and EC0880B are available under ENA accessions ERR3142418 and ERR3142524, respectively.

**Impact Statement**

Nitrofurantoin is widely used for treating and preventing urinary tract infection (UTI). The prevalence of nitrofurantoin resistance is generally low in *Escherichia coli *. Our work discovered nitrofurantoin heteroresistance in distinct *E. coli* clinical isolates and attributed this phenotype to IS*1*-associated deletion of chromosomal regions harbouring the oxygen-insensitive nitroreductase gene *nfsB* in a genetic background where *nfsA* was inactivated. Our findings demonstrate a novel genetic mechanism of nitrofurantoin heteroresistance and suggest surveillance for this phenotype in *E. coli* urinary isolates for improving UTI management.

## Introduction

Nitrofurantoin is a widely used first-line antimicrobial for treatment and prophylaxis of urinary tract infection (UTI) [1]. Reduced nitrofurantoin susceptibility in *Escherichia coli* has been associated with inactivating mutations in chromosomal genes *nfsA*, *nfsB* and *ribE* [2], which encode key components of the oxygen-insensitive nitroreductase system, and with an acquired multidrug efflux pump OqxAB [3]. Despite low prevalence of nitrofurantoin resistance in *E. coli* clinical isolates (<7 % in Europe) [4, 5], concerns over increased prevalence of nitrofurantoin resistance are growing in England, where the consumption of nitrofurantoin had increased by 41 % from 2017 to 2021 [6].

Studies have reported that *E. coli* clinical isolates may show heteroresistance against polymyxins and carbapenems [7], a phenomenon whereby subpopulations within an isolate demonstrate lower susceptibility to a specific antimicrobial (hereafter, a resistant subpopulation) than the main population [7–9]. Nitrofurantoin heteroresistance in clinical isolates of *E. coli* or other species had not been reported until our recent observation of potential examples in two *E. coli* blood isolates [10]. We therefore set out to confirm nitrofurantoin heteroresistance and determine relevant genetic mechanisms for both isolates.

## Methods

### *E. coli* isolates

Two *E. coli* isolates, EC0026B (SAMEA104039660) and EC0880B (SAMEA104040147), of Achtman multi-locus sequence types ST484 and ST58, respectively, were previously collected from independent bloodstream infections [11] and were tested in this study to confirm nitrofurantoin heteroresistance. A nitrofurantoin-resistant isolate IN09 [10] and nitrofurantoin-susceptible strain ATCC 25922 [12] were included as positive and quality controls, respectively, for population analysis profiling (PAP), which is the gold standard for detecting antimicrobial heteroresistance [8]. Illumina whole-genome sequencing (WGS) reads of EC0026B and EC0880B have been generated previously [11] and are available under accessions ERR3142418 and ERR3142524, respectively, in the ENA. We have shown previously that each of EC0026B and EC0880B carries a loss-of-function mutation in the *nfsA* gene [10].

### Population analysis profiling

**Fig. 1.**
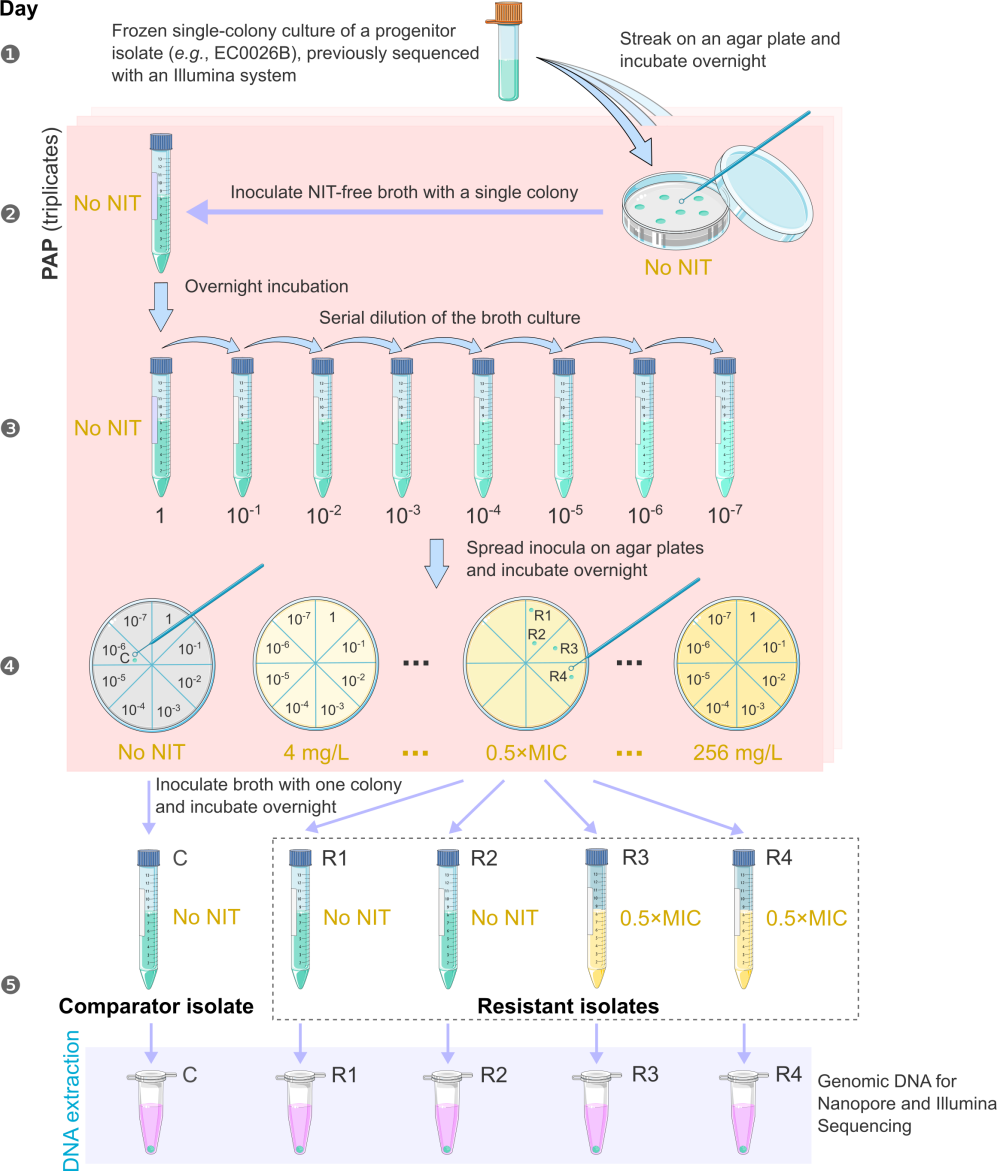
Workflow of population analysis profiling for each progenitor *E. coli* isolate and preparation of isolates for DNA extraction. Resistant isolates R1–R4, which showed reduced nitrofurantoin (NIT) susceptibility when compared to the majority of cells in the progenitor isolate, were randomly chosen on Day 4. Isolate C is a comparator, which underwent the same number of passages as resistant isolates. NIT concentrations in culture media are noted in gold. Icons were downloaded from Bioicons (bioicons.com) under a CC-BY 3.0 Licence.

Nitrofurantoin-resistant subpopulations of each isolate (EC0026B, EC0880B, IN09 and ATCC 25922) were sought in three independent PAP experiments to confirm reproducibility (Fig. 1). Specifically, nitrofurantoin (N7878; Sigma-Aldrich) was dissolved in *N*,*N*-dimethylformamide (494488; Sigma-Aldrich) to produce a stock solution of 50 mg ml^-1^ nitrofurantoin, which was then infused into cation-adjusted Mueller-Hinton (MH2; 90922; Sigma-Aldrich) agar to form a series of two-fold increments in nitrofurantoin concentrations (4–256 mg l^−1^). One colony of each isolate was inoculated into nitrofurantoin-free MH2 broth and grown aerobically overnight at 37 °C with shaking (180 r.p.m.). Seven serial 10-fold dilutions of each broth culture were created with PBS (P4417; Sigma-Aldrich). Aliquots (10 µl) of the original broth culture and serial dilutions were spread in octants of MH2 agar plates following the nitrofurantoin-concentration gradient and incubated aerobically overnight at 35 °C. For each agar plate, colony-forming units were counted in the octant where colonies were well separated, and thereby the nitrofurantoin minimum inhibitory concentration (MIC) of each isolate was determined. A maximum non-inhibitory nitrofurantoin concentration of each isolate was defined as the greatest concentration at which ≥70 % cells grew when compared to the growth without nitrofurantoin.

### Deriving isolates from progenitor isolates EC0026B and EC0880B

Following PAP assays and for each of EC0026B and EC0880B, four colonies growing on agar containing 0.5× MIC nitrofurantoin (*e.g.* 32 mg l^−1^, if the isolate’s MIC was 64 mg l^−1^) were randomly chosen as resistant isolates (denoted by a subscript ‘R’, *e.g.* EC0026B_R1_). To extract DNA and compare isolates with or without an extended time of nitrofurantoin exposure, these resistant isolates were divided into two groups (two isolates per group) for inoculating MH2 broth with (0.5× MIC) or without nitrofurantoin and aerobically incubated overnight at 35 °C (Fig. 1). From the same PAP experiment where the four resistant isolates of each progenitor isolate were derived (at 0.5× MIC nitrofurantoin), an additional colony was randomly selected from a nitrofurantoin-free agar plate as a comparator isolate (denoted by subscript ‘C’, *e.g.* EC0026B_C_) and was grown in nitrofurantoin-free MH2 broth before DNA extraction (Fig. 1). Therefore, the comparator isolate had never been exposed to nitrofurantoin.

### DNA extraction and whole-genome sequencing

For resistant and comparator isolates derived from isolates EC0026B and EC0880B, genomic DNA was extracted from the broth culture using proteinase K solution, RNase A solution and cell lysis solution (Qiagen), and purified using a GeneJET Genomic DNA Purification Kit (ThermoFisher Scientific). The mean concentration of DNA in each extract was estimated from three reads obtained with a Qubit dsDNA BR Assay Kit (ThermoFisher Scientific).

Extracted DNA of each isolate was aliquoted for WGS. Short-read sequencing (101 bp, paired-end) was conducted on an Illumina HiSeq 2500 system (Illumina), and long-read sequencing was conducted on a MinION R9.4.1 flow cell (Oxford Nanopore Technologies) for isolates having adequate yields of DNA. For MinION sequencing, DNA libraries were prepared using a Rapid Barcoding Kit SQK-RBK004 (Oxford Nanopore Technologies), and base-calling, demultiplexing and barcode trimming were conducted with Guppy v5.0.16 (community.nanoporetech.com/downloads) and its built-in high-accuracy model. For quality control, Illumina reads were trimmed for a minimal base quality of Phred Q20 (10 bp sliding window) and filtered for a minimal length of 50 bp using Trimmomatic [13]; MinION reads were filtered for a minimal per-read average quality of Q10 and minimal length of 1 kb using NanoFilt v2.8.0 [14].

### Genome assembly and annotation

For isolates having MinION and Illumina reads, MinION reads were assembled *de novo* using Raven v1.7.0 [15] and polished with the same reads four times by Raven and for an additional round by Medaka v1.7 (github.com/nanoporetech/medaka); for isolates only having Illumina reads, genomes were assembled using Unicycler v0.5.0 [16]. All assemblies were then polished with Illumina reads using Polypolish v0.5.0, POLCA v4.0.9 and again Polypolish [17–19]. Complete chromosome and plasmid assemblies were rotated to start from *dnaA* and *rep* genes, respectively. Scripts used for aforementioned steps are available at github.com/wanyuac/Assembly_toolkit. Genome annotation was conducted with Prokka v1.14.6 [20]. Insertion sequences were inferred from annotations and searched against the ISFinder database [21] for confirmation.

### Variant identification

For isolates derived from the same progenitor, the complete chromosome sequence of the comparator isolate was used as a reference to identify chromosomal genetic variation in resistant isolates. Since chromosomes of isolates having MinION reads were fully assembled, isolate-specific alignments of these chromosomes against references were performed using Minimap2 v2.24 [22]. Chromosomal mutations and structural variations were identified from alignments using paftools.js of Minimap2. For isolates only having Illumina reads, chromosomal mutations were identified from isolate-specific read mapping using Minimap2, Samtools v1.16.1 and BCFtools v1.16 (DP≥10, QUAL≥20 and MQ≥30) [23], and structural variations were determined with breseq v0.37.1 [24].

For each isolate, mutations were filtered to exclude those in repetitive chromosomal regions (≥90 % nucleotide identity, determined in the reference with MUMmer v4.0.0rc1) [25] for accuracy and then mapped against annotations of the reference sequence with SnpEff v4.3.1t to estimate functional impacts [26]. Mutations and structural variations were inspected in sequence alignments using Artemis v18.2.0 [27], and genetic structures were illustrated using BRIG v0.95 [28] and the R package gggenes (wilkox.org/gggenes).

## Results

### Phenotypic confirmation of nitrofurantoin heteroresistance

**Fig. 2.**
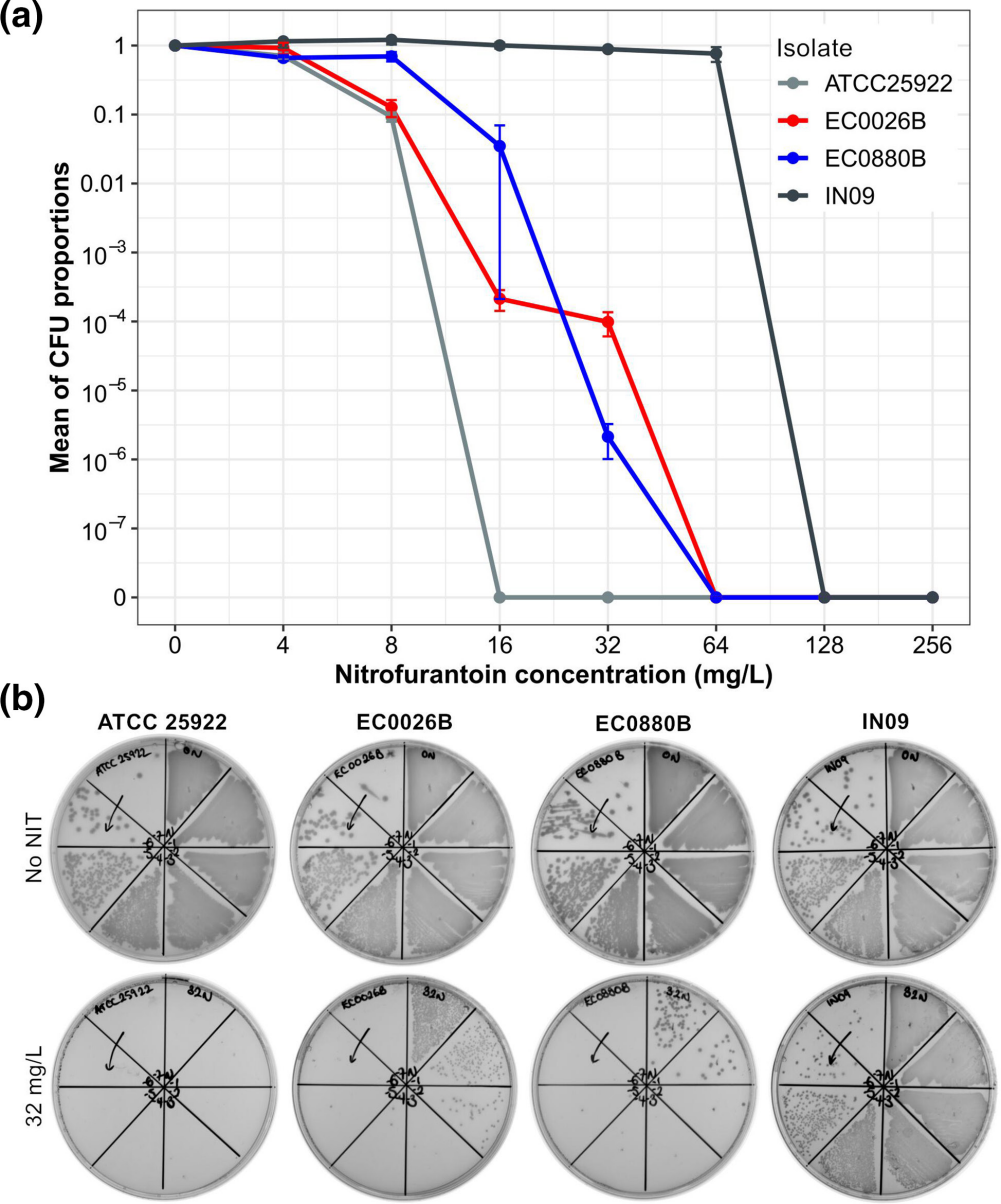
Results of population analysis profiling (PAP). (**a**) PAP curves of test and control *E. coli* isolates. Test isolates EC0026B and EC0880B demonstrate nitrofurantoin (NIT) heteroresistance. Control isolate IN09 is known to be NIT-resistant, and ATCC 25922 is NIT-susceptible. For each isolate, the proportion of colony-forming units (CFUs) growing at each NIT concentration (4256 mg l^−1^) was calculated by dividing the number of CFUs with that of the inoculum CFUs (counted from the NIT-free agar plate). Based on biological triplicates, the mean±sem proportion of CFUs is shown. CFU counts below the detection limit are denoted by ‘0’ on the *y*-axis. (**b**) Growth of test and control isolates on cation-adjusted Mueller-Hinton agar plates with or without NIT in the same experiment. Test isolates EC0026B and EC0880B showed subpopulations that were able to grow at the NIT concentration of 32 mg l^−1^.

PAP assays confirmed nitrofurantoin heteroresistance in both *E. coli* blood isolates EC0026B and EC0880B. Each heteroresistant isolate had a nitrofurantoin MIC of 64 mg l^−1^, which was 8–16 times its maximum non-inhibitory concentration (EC0026B: 4 mg l^−1^; EC0880B: 8 mg l^−1^; Fig. 2). The average proportion of colony-forming units able to grow at 32 mg l^−1^ nitrofurantoin was 9.85×10^−5^ and 2.14×10^−6^ for EC0026B and EC0880B, respectively. In comparison, the MIC of the control strain ATCC 25922 (16 mg l^−1^) was four times its maximum non-inhibitory concentration, and positive control IN09 (MIC=128 mg l^−1^) showed a drastic transition from full growth to complete inhibition when the nitrofurantoin concentration doubled from 64 mg l^−1^.

### *E. coli* isolates and genome assemblies

Four resistant isolates and one comparator isolate were derived from each of isolates EC0026B and EC0880B (Fig. 1), providing 10 isolates altogether for WGS. Complete genomes were assembled for nine of these isolates, and only a draft genome assembly was available for resistant isolate EC0880B_R1_, since its DNA yield was not adequate for MinION sequencing (Table S2).

### Genetic variation in isolates derived from isolate EC0026B

**Fig. 3.**
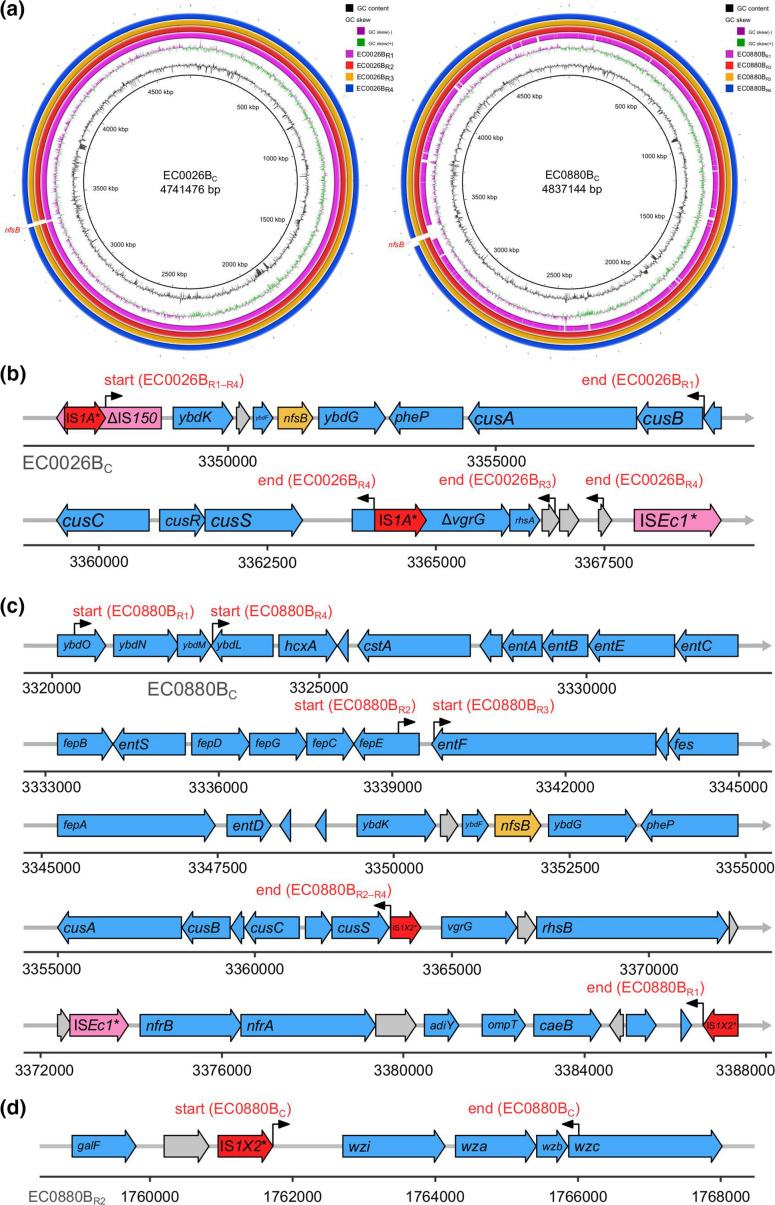
Genetic structures of deleted regions. (**a**) BRIG plots comparing genome assemblies against chromosome sequences of EC0026B_C_ and EC0880B_C_. Sequences were aligned using megaBLAST, and matches were filtered for a minimum nucleotide identity of 90 % and a minimum query coverage of 0.6 %. (b, c) Genetic structures of *nfsB*-carrying regions deleted from the chromosomes of EC0026B_C_ and EC0880B_C_, respectively, as indicated in (a). Start and end positions of each deletion are indicated by black arrows and red labels. Asterisks indicate variants of insertion sequences against reference sequences from the ISFinder database, and grey wide arrows indicate coding sequences of hypothetical proteins. (**d**) Genetic structure of the capsule-encoding region in the chromosome of EC0880B_R2_ and the partial deletion of this region in EC0880B_C_.

Resistant isolates EC0026B_R1–R4_ showed different deletions of 11–20 kb chromosomal regions harbouring *nfsB* (Fig. 3a), and each deletion immediately followed the right imperfect inverted repeat (IRR) of an IS*1*-family insertion sequence IS*1A*, which interrupted insertion sequence IS*150* (Fig. 3b). The other end of the deletion seemed variable and occurred in coding sequences. For instance, the deletion in the EC0026B_R4_ chromosome ended 10 bp upstream of the left imperfect inverted repeat (IRL) of another IS*1A*, which interrupted the gene *vgrG*. These two IS*1A* elements and the relevant reference sequence in the ISFinder database mutually differed by two nucleotide substitutions in the second open reading frame (*insB*) of the IS*1A* transposase gene [29].

Twenty-four copies of IS*1A* were found in chromosomes of EC0026B_C_, EC0026B_R1_ and EC0026B_R4_, and 23 copies were found in EC0026B_R2_ and EC0026B_R3_, which is consistent with deletions illustrated in Fig. 3b. These IS*1A* elements demonstrated 99–100% nucleotide identities and a 100 % coverage to the reference IS*1A* sequence in the ISFinder database. The explicit copy number of IS*1A* could not be determined in the Unicycler short-read-only assembly of EC0026B. Nevertheless, in the assembly graph, the mean and median depths of assembled segments from which the complete IS*1A* was recovered suggest 20–22 copies of this element with >98 % nucleotide identities to the reference sequence.

As for other chromosomal genetic variation, 14 nucleotide substitutions and two indels were found in EC0026B_R1_, whereas no point mutation was found in EC0026B_R2_, EC0026B_R3_, or EC0026B_R4_ (Table 1). No genetic variation was detected between EC0026B_C_ and EC0026B.

**Table 1.** Genetic variation in resistant isolates EC0026B_R1–R4_ when compared to the chromosome of EC0026B_C_.

Isolate	Position	DNA change	Gene	Protein change
EC0026B_R1_	449 628	T>C	*chiA*	S779P
	461 138	A>G	*gspC*	None (synonymous)
	603693–603 694	Insertion: 78 bp	*deaD*	G585(26 aa)R586
	1 417 135	C>T	–	None (intergenic)
	1 705 072	A>G	*dld*	None (synonymous)
	1 962 017	T>C	*uvrY*	L176P
	1 977 043	Deletion: T	*uspC*	Q18fs
	2 128 454	T>G	*astD*	None (synonymous)
	2 355 150	C>T	Hypothetical	V106I
	2 895 745	G>A	*yccM*	A47T
	3 158 149	T>C	*ybhI*	None (synonymous)
	3 206 276	T>C	*gltA*	V144A
	3 207 019	T>C	*gltA*	W392R
	3347713–3 358 894	Deletion (11 kb)	Multiple	Loss of protein products
	3 638 980	A>G	Hypothetical	T69A
	3 753 165	G>T	*aacA*	None (synonymous)
	3 784 606	C>T	Hypothetical	S348N
EC0026B_R2_	3347713–3 367 484	Deletion (20 kb)	Multiple	Loss of protein products
EC0026B_R3_	3347713–3 366 768	Deletion (19 kb)	Multiple	Loss of protein products
EC0026B_R4_	3347713–3 364 083	Deletion (16 kb)	Multiple	Loss of protein products

aa, amino acid.

### Genetic variation in isolates derived from isolate EC0880B

Resistant isolates EC0880B_R1–R4_ showed different large deletions (24–66 kb) of chromosomal regions harbouring *nfsB* (Fig. 3a and Table 2). All these deletions ended immediately upstream of two identical copies of an IS*1*-family insertion sequence IS*1X2* (Fig. 3c). However, the start site of each deletion was variable. This IS*1X2* element showed a 98 % nucleotide identity and 100 % coverage to its reference sequence in the ISFinder database.

**Table 2.** Genetic variation in resistant isolates EC0880B_R1–R4_ when compared to the chromosome of EC0880B_C_. Of note, the 4.3 kb ‘insertion’ resulted from the deletion of this region in the chromosome of EC0880B_C_.

Isolate	Position	DNA change	Gene	Protein change
EC0880B_R1_	1761728–1 761 729	‘Insertion’ (4.3 kb)	*wzi, a, b, c*	No change
	3320419–3 386 613	Deletion (66 kb)	Multiple	Loss of protein products
EC0880B_R2_	1 736 645	A>T	*mdtC*	M284L
	1761728–1 761 729	‘Insertion’ (4.3 kb)	*wzi, a, b, c*	No change
	3 317 674	G>T	*ahpF*	Q147H
	3339108–3 363 443	Deletion (24 kb)	Multiple	Loss of protein products
	4 117 518	A>C	*uxuB*	N187H
EC0880B_R3_	1761728–1 761 729	‘Insertion’ (4.3 kb)	*wzi, a, b, c*	No change
	3339723–3 363 443	Deletion (24 kb)	Multiple	Loss of protein products
EC0880B_R4_	1761728–1 761 729	‘Insertion’ (4.3 kb)	*wzi, a, b, c*	No change
	3323004–3 363 443	Deletion (40 kb)	Multiple	Loss of protein products

Unexpectedly, an additional chromosomal 4289 bp deletion was identified in EC0880B_C_ when compared with the genome assembly of EC0880B despite absence of any point mutations. This deleted region was present in EC0880B_R1–R4_ (Table 2) and immediately followed the IRR of the same IS*1X2* as those related to the deletion of *nfsB* (Fig. 3d). This deletion caused a loss of three genes (*wzi*, *wza* and *wzb*) and a 142 bp truncation of the 5′ end of *wzc* in the conserved capsule locus *wzi-wza-wzb-wzc* [30].

Twelve copies of IS*1X2* were found in chromosomes of EC0880B_C_, EC0880B_R2_, EC0880B_R3_ and EC0880B_R4_, with 96–98% nucleotide identities and a 100 % coverage to the reference IS*1X2* sequence. The precise copy number of IS*1X2* could not be determined in the Unicycler short-read-only assembly of EC0880B_R1_, although 100 % of this insertion sequence was recovered from five connected nodes (with 96–100% nucleotide identities to the reference sequence) in the assembly graph of this genome. These five nodes had a median read depth of 11-fold, suggesting 11 copies of IS*1X2*, which is consistent with the identified deletion in this isolate’s chromosome (Fig. 3c). Similarly, 11 copies of IS*1X2* were estimated from the Unicycler short-read-only assembly graph of EC0880B with ≥95 % nucleotide identities.

Interestingly, the other oxygen-insensitive nitroreductase gene *nfsA* in chromosomes of EC0880B_C_, EC0880B_R2_, EC0880B_R3_ and EC0880B_R4_ was interrupted by the same IS*1X2* element that was associated with the deletion of *nfsB*, and locations of these interruptions were the same as that previously observed in EC0880B [10]. The same interruption of *nfsA* was seen in EC0880B_R1_, although the sequence of the interruptive IS*1X2* could not be reliably recovered from the short-read-only assembly graph of this genome.

## Discussion

We confirmed nitrofurantoin heteroresistance in *E. coli* blood isolates EC0026B and EC0880B through PAP assays. The MIC of each isolate was 8–16 times its maximum non-inhibitory nitrofurantoin concentration, and each isolate had a small subpopulation (2.14×10^−6^–9.85×10^−5^ c.f.u.) that could grow at 32 mg l^−1^ (0.5× MIC) nitrofurantoin. The resistant subpopulation in each nitrofurantoin-heteroresistant isolate was more frequent than a minimum of 10^−7^ proposed to define heteroresistance [7] but may not be detectable as a skipped-well phenomenon using the reference broth microdilution method (ISO 20776-1 : 2019) [31, 32]. Since *nfsA* and *ribE* were identical between each progenitor isolate (EC0026B or EC0880B) and its derived resistant and comparator isolates, and neither progenitor isolate produces the multidrug-efflux pump OqxAB [10], we attribute the observed nitrofurantoin heteroresistance to the IS*1*-associated deletion of *nfsB* regions. This kind of deletion probably occurred in a UTI-associated *E. coli* ST131 strain, whose nitrofurantoin MIC increased from 8 to 128 mg l^−1^ upon intermittent *in vivo* exposures to a therapeutic dose of nitrofurantoin in six months [33, 34].

We also show that an IS*1* element, IS*1X2*, was associated with the truncation of capsule locus *wzi-wza-wzb-wzc* in EC0880B_C_. All deletion events observed in our study are consistent with the abortive transposition model of IS*1*-mediated deletion, where a DNA duplex nicks at an end of IS*1* and ligates to a target site on the same molecule, causing a loss of DNA between these two breakpoints [35]. Moreover, IS*1X2* had interrupted *nfsA* in EC0880B, and we have previously elucidated that IS*1R* interrupted *nfsA* in two nitrofurantoin-resistant isolates IN01 and IN02 [10]. Taken together, IS*1*-associated deletion and interruption of *nfsA* or *nfsB* reduces nitrofurantoin susceptibility of *E. coli* and such genetic variation may occur at other loci, potentially altering phenotypes. As such, we emphasise the importance of monitoring the prevalence and genomic locations of IS*1* using long-read sequencing.

Nitrofurantoin heteroresistance in *E. coli* strain K-12 MG1655 was reported previously, with a small subpopulation growing at a maximum nitrofurantoin concentration of 8 mg l^−1^ while the majority of cells had an MIC of 4 mg l^−1^ [36]. Our PAP experiment discovered similar heteroresistance in *E. coli* strain ATCC 25922, which showed a maximum non-inhibitory nitrofurantoin concentration of 4 mg l^−1^ and a subpopulation growing at 8 mg l^−1^ with an average frequency of 9 % (Fig. 2a). Nonetheless, neither the level of the reduction in nitrofurantoin susceptibility nor the nitrofurantoin MIC of each isolate is as great as those of isolates EC0026B and EC0880B. Furthermore, the inactivating mutations in *nfsA* may have been prerequisites for the level of resistance in both heteroresistant isolates (nitrofurantoin MIC=64 mg l^−1^), since loss of *nfsA* or *nfsB* function alone only has limited (MIC ≤32 mg l^−1^) or no impact on the nitrofurantoin susceptibility of *E. coli* [10, 33].

Since nitrofurantoin MICs of both EC0026B and EC0880B were at the clinical breakpoint for nitrofurantoin resistance (>64 mg l^−1^) determined by the European Committee on Antimicrobial Susceptibility Testing [37], both isolates may grow under therapeutic or prophylactic dosing of nitrofurantoin [38, 39] and gain adaptive mutations under sub-MIC exposure [40] in the urinary tract or gut. Moreover, the deletion of *nfsB* in these isolates, which already had inactivating mutations in *nfsA*, would cause an irreversible reduction in their nitrofurantoin susceptibility, paving the way to the emergence of nitrofurantoin-resistant cells if there were to be subsequent resistance mutations, such as those in *ribE* and the *marA-marB* intergenic region [40, 41]. Importantly, however, because the loss of *nfsB* reduces the reproduction rate of *E. coli* [33], the establishment of *ΔnfsB* mutants may only occur under selective pressure of nitrofurantoin.

We consider IS*1*-associated nitrofurantoin heteroresistance to be a potential threat to the management of UTI for three reasons. First, the frequency of the resistant subpopulation in a heteroresistant isolate exceeds the frequency of spontaneous resistance point mutations occurring in a susceptible isolate. For instance, the frequency of the subpopulation in EC0026B and EC0880B growing at 32 mg l^−1^ nitrofurantoin (from 2 to 99 c.f.u. per million) was 10–100 times the reported frequencies of spontaneous resistance point mutations in *E. coli* [42, 43]. An early study shows a 30- to 2000-fold increase in the deletion frequency when IS*1* is present [44]. Second, IS*1* provides *E. coli* with adaptive fitness. Specifically, in the absence of nitrofurantoin, the susceptible major population in an IS*1*-related nitrofurantoin-heteroresistant isolate can transmit and establish colonisation or infection as homogeneously nitrofurantoin-susceptible *E. coli* does and probably outcompete nitrofurantoin-resistant *E. coli* that has lost functions of *nfsA* and/or *nfsB*; and in the presence of nitrofurantoin, the infection/colonisation may persist with the resistant subpopulation. Third, nitrofurantoin-heteroresistant isolates may not be detected by routine nitrofurantoin-susceptibility tests in clinical settings if the resistant subpopulation has a frequency below the test’s detection limit.

To determine the mechanism of nitrofurantoin heteroresistance in *E. coli *, our study took advantage of hybrid *de novo* assemblies in accurately resolving complete bacterial genomes and identifying structural variation. Further work is needed to elucidate how IS*1* is associated with genetic deletions and to compare fitness between resistant mutants and heteroresistant progenitor isolates. Moreover, since this work focused on two *E. coli* blood isolates in which gene-deletion events were associated with different IS*1* variants, the prevalence of IS*1*-associated nitrofurantoin heteroresistance and genomic rearrangements in *E. coli* have yet to be fully assessed within a wider strain collection including urinary isolates. This future work would determine whether gene deletions require specific combinations of isolates and insertion sequences, as might be inferred from the isolate-specific nucleotide substitutions and indels in resistant isolates derived from EC0026B and EC0880B (Tables 1 and 2). Finally, heightened surveillance for prevalence and insertion sites of IS*1* in clinical *E. coli* isolates will help us to understand how this insertion sequence contributes to bacterial evolution.

## Conclusions

*E. coli* can be stably heteroresistant to nitrofurantoin, with potential clinical significance, and such heteroresistance may be a precursor to nitrofurantoin resistance. IS*1*-family elements are associated with large-scale deletion of genomic regions containing *nfsB* and are potentially important agents of genomic and phenotypic changes in *E. coli *.

## Supplementary Data

Supplementary material 1
