# Supplementary material for: IS1-related large-scale deletion of chromosomal regions harbouring the oxygen-insensitive nitroreductase gene nfsB causes nitrofurantoin heteroresistance in Escherichia coli
Source: Microb Genom. 2023 Sep 6;9(9):001102. doi: 10.1099/mgen.0.001102 (PMC10569738; doi:10.1099/mgen.0.001102)
Supplement: Supplementary material 1 [file mgen-9-1102-s001.pdf]

# IS*1*-related large-scale deletion of chromosomal regions harbouring the oxygen-insensitive nitroreductase gene *nfsB* causes nitrofurantoin heteroresistance in *Escherichia coli*

## Supplementary tables

Yu Wan, Akshay Sabnis, Zaynab Mumin, Isabelle Potterill, Elita Jauneikaite, Colin S. Brown, Matthew J. Ellington, Andrew Edwards, and Shiranee Sriskandan

August 2023

**Table S1.** Accession numbers of *E. coli* whole-genome sequencing data deposited under BioProject PRJEB58678 in the European Nucleotide Archive (www.ebi.ac.uk/ena).

| Progenitor | Isolate | BioSample | Illumina reads | MinION reads | Assembly (GenBank) |
|---|---|---|---|---|---|
| EC0026B | EC0026B$_{C}$ | SAMEA112856587 | ERR11181256 | ERR11181589 | OX460329 – OX460332 |
| | EC0026B$_{R1}$ | SAMEA112856588 | ERR11181257 | ERR11181590 | OX460336 – OX460339 |
| | EC0026B$_{R2}$ | SAMEA112856589 | ERR11181258 | ERR11181591 | OX460326 – OX460328 |
| | EC0026B$_{R3}$ | SAMEA112856590 | ERR11181259 | ERR11181592 | OX460323 – OX460325 |
| | EC0026B$_{R4}$ | SAMEA112856591 | ERR11181260 | ERR11181593 | OX460316 – OX460318 |
| EC0880B | EC0880B$_{C}$ | SAMEA112856592 | ERR11181261 | ERR11181594 | OX460340 – OX460342 |
| | EC0880B$_{R1}$ | SAMEA112856593 | ERR11181262 | Not applicable | CATKUL010000000 |
| | EC0880B$_{R2}$ | SAMEA112856594 | ERR11181263 | ERR11181595 | OX460333 – OX460335 |
| | EC0880B$_{R3}$ | SAMEA112856595 | ERR11181264 | ERR11181596 | OX460319 – OX460322 |
| | EC0880B$_{R4}$ | SAMEA112856596 | ERR11181265 | ERR11181597 | OX460313 – OX460315 |

**Table S2.** Summary of *E. coli* isolates and complete sequences (all circular) recovered from *de novo* genome assemblies. Plus signs indicate isolates for which MinION reads were available, and asterisks indicate chromosome sequences used as references for genomic comparison. Complete chromosome sequence of EC0880B$_{R1}$ could not be recovered because of inadequate DNA extracted from this isolate for MinION sequencing.

| Progenitor | Isolate | MinION | Chromosome length | Plasmid length |
|---|---|---|---|---|
| EC0026B | EC0026B$_{C}$ | + | 4,741,476 bp * | 88,489 bp; 73,423 bp; 1,788 bp |
| | EC0026B$_{R1}$ | + | 4,730,372 bp | 88,654 bp; 73,432 bp; 1,787 bp |
| | EC0026B$_{R2}$ | + | 4,721,705 bp | 88,510 bp; 73,423 bp |
| | EC0026B$_{R3}$ | + | 4,722,421 bp | 88,442 bp; 73,423 bp |
| | EC0026B$_{R4}$ | + | 4,725,107 bp | 88,429 bp; 73,423 bp |
| EC0880B | EC0880B$_{C}$ | + | 4,837,144 bp * | 159,448 bp; 33,743 bp |
| | EC0880B$_{R1}$ | | Not recovered | 33,743 bp; 1,783 bp |
| | EC0880B$_{R2}$ | + | 4,817,091 bp | 159,448 bp; 33,743 bp |
| | EC0880B$_{R3}$ | + | 4,817,712 bp | 159,448 bp; 33,743 bp; 1,783 bp |
| | EC0880B$_{R4}$ | + | 4,800,993 bp | 159,448 bp; 33,743 bp |
